# Pathological Comparisons of the Hippocampal Changes in the Transient and Permanent Middle Cerebral Artery Occlusion Rat Models

**DOI:** 10.3389/fneur.2019.01178

**Published:** 2019-11-14

**Authors:** Fawad Ali Shah, Tao Li, Lina Tariq Al Kury, Alam Zeb, Shehla Khatoon, Gongping Liu, Xifei Yang, Fang Liu, Huo Yao, Arif-Ullah Khan, Phil Ok Koh, Yuhua Jiang, Shupeng Li

**Affiliations:** ^1^State Key Laboratory of Oncogenomics, School of Chemical Biology and Biotechnology, Peking University Shenzhen Graduate School, Shenzhen, China; ^2^Department of Pharmacology, Riphah Institute of Pharmaceutical Sciences, International University, Islamabad, Pakistan; ^3^Department of Forensic Medicine, School of Medicine, Xi'an Jiaotong University, Xi'an, China; ^4^College of Natural and Health Sciences, Zayed University Abu Dhabi, Abu Dhabi, United Arab Emirates; ^5^Department of Anatomy, Khyber Medical College, Khyber Medical University, Peshawar, Pakistan; ^6^Department of Pathophysiology, School of Basic Medicine and the Collaborative Innovation Center for Brain Science, Key Laboratory of Ministry of Education of China and Hubei Province for Neurological Disorders, Tongji Medical College, Huazhong University of Science and Technology, Wuhan, China; ^7^Co-innovation Center of Neuroregeneration, Nantong University, Nantong, China; ^8^Key Laboratory of Modern Toxicology of Shenzhen, Shenzhen Center for Disease Control and Prevention, Shenzhen, China; ^9^Department of Psychiatry, University of Toronto, Toronto, ON, Canada; ^10^Centre for Addiction and Mental Health, Campbell Research Institute, Toronto, ON, Canada; ^11^Department of Anatomy, College of Veterinary Medicine, Research Institute of Life Science, Gyeongsang National University, Jinju-si, South Korea; ^12^Cancer Centre, The Second Hospital of Shandong University, Jinan, China

**Keywords:** ischemic stroke, hippocampus, diabetes, transient and permanent cerebral ischemia, neurodegeneration, glutamate receptor, inflammation, ROS

## Abstract

Ischemic strokes are categorized by permanent or transient obstruction of blood flow, which impedes delivery of oxygen and essential nutrients to brain. In the last decade, the therapeutic window for tPA has increased from 3 to 5–6 h, and a new technique, involving the mechanical removal of the clot (endovascular thrombectomy) to allow reperfusion of the injured area, is being used more often. This last therapeutic approach can be done until 24 h after stroke onset. Due to this fact, more acute ischemic stroke patients are now being recanalized, and so tMCAO is probably the “best” model to address these patients that have a potential good outcome in terms of survival and functional recovery. However, permanent occlusion patients are also important, not only to increase survival rate but also to improve functional outcomes, although these are more difficult to achieve. So, both models are important, and which target different stroke patients in the clinical scenario. Hippocampus has a vital role in memory and cognition, is prone to ischemic induced neurodegeneration. This study was designed to delineate the molecular, pathological, and neurological changes in rat models of t-MCAO, permanent MCAO (pMCAO), and pMCAO with diabetic conditions in hippocampal tissue. Our results showed that these three models showed distinct discrepancies at numerous pathological process, including key signaling molecules involved in neuronal apoptosis, glutamate induced excitotoxicity, neuroinflammation, oxidative stress, and neurotrophic changes. Our result suggests that the two commonly used MCAO models exhibited tremendous differences in terms of neuronal cell loss, glutamate excitotoxic related signaling, synaptic transmission markers, neuron inflammatory and oxidative stress molecules. These differences may reflect the variations in different models, which may provide valuable information for mechanistic and therapeutic inconsistences as experienced in both preclinical models and clinical trials.

## Introduction

Stroke accounts for great number of death and disability across the globe. The frequency of stroke varies with demographic location, and generally considered to be the 2nd leading cause of mortality in industrialized countries. Ischemic stroke represents the frequently encounter stroke type, caused by thrombosis or dislodged emboli. The characteristic events in ischemic stroke include extensive depolarization, release of excitatory glutamate, opening of voltage gated ion channels, and intracellular Ca^2+^ buildup. Induced by intraluminal suture, focal cerebral ischemia is a well-established animal stroke model of clinical relevance, comprising of transient (t-MCAO) and permanent (p-MCAO). t-MCAO is extensively employed in about 88% basic experimental MCAO models from 2014 to 2015 (1). Despite of several advantages (2–4), many consistent studies suggested some shortfall to t-MCAO (5, 6). Contrary, p-MCAO mimics the large vessel occlusion (LVO) due to no reperfusion process, and is equally important. However, permanent occlusion is more difficult to achieve and it is associated with increase mortality attributed to higher swelling and intracranial pressure. So, both models are important, and target different stroke patients in the clinical scenarios.

Pathological changes of both t-MCAO and p-MCAO models included core region where blood flow dropped from 10 to 25% and is composed of neuronal necrosis, surrounded by penumbra region where brain tissue suffered from mild to moderate ischemic damage. The infarcted region in rat comprised primarily of cortex, striatum, thalamus and hypothalamus, while in mice the infarction also extent to hippocampus (7, 8). Moreover, sensitivity of different brain regions to ischemia varies based on collateral circulation, rodent strain, and experimental set up such as t-MCAO and p-MCAO (9–11). Many studies confined ischemic core to striatum when MCA occluded for 30 min, increasing ischemic interval will proportionally expends core and penumbral boundaries (12, 13). As a result, t-MCAO and p-MCAO models represented different advantages coherent to both the clinical scenario and underlying mechanisms.

There are limited studies on hippocampus as per our information and literature study using permanent MCAO (p-MCAO) approach. Few studies demonstrated morphological and biochemical variations in hippocampus following p-MCAO (14–16) The hippocampus in rodents is supplied by anterior choroidal artery (a branch form ICA) and posterior hippocampal artery (branch arising from posterior communication artery, PcomA) (17). As such, the intraluminal blockage will hinder blood supply in choroidal artery, with minimum effect on flow in posterior hippocampal artery. Moreover, various factors influencing the extent of ischemic stroke, such as suture/nylon filament diameter (3–0, 4–0), length of filament, coating by (silicone or poly-L-lysine), and tip of filament (18–20). Among, duration of artery occlusion has a more pronounced effect on volume of infarction (12, 13).

In the present study, we have compared the molecular changes in rat models of t-MCAO, p-MCAO, and p-MCAO with diabetic condition in hippocampus. Diabetes is an independent risk factor and important comorbid of stroke, which not only increased the risk of ischemic stroke to 1.5–3 times, but also worsened the stroke outcomes with increased aggravated neurological deficits, functional disabilities, and mortality (21, 22). Our objective is to determined hippocampus response after transient and permanent ischemia and secondly to delineate inconsistencies in neuronal apoptosis, excitotoxicity, neuroinflammation, and neurogenesis in rat models of t-MCAO, p-MCAO, and p-MCAO with diabetic condition as these discrepancies may partially explain repetitive failures of experimental findings during clinical trials. Thus, further characterizing the detailed molecular and cellular processes will unveil the complex pathological processes and will provide the basis for more coherent clinical interventions.

## Experimental Procedures

### Animals Grouping and Drug Treatment

Adult male Sprague–Dawley rats weighing 200–230 g (8–9 weeks) were purchased from Guangdong Medical Laboratory Animal Center, China. The experimental animals were housed at Laboratory Animal Research Center, Peking University Shenzhen Graduate School, under 12 h light/12 h dark cycle at 18–22°C and had free access to diet and tap water throughout the study. The experimental procedures were set in such a way to minimize rats suffering. All experimental procedures were carried out according to the protocols approved by Institutional Animal Care and Use Committee of Peking University Shenzhen Graduate School. We did not use any blind allocation or randomization of rats, instead we adhered the criteria to keep similar weight animal to same group under the same experimental condition. The rats were randomly divided into 4 groups (*n* = 15/group) containing: Sham operated control group; transient middle cerebral artery occlusion for 90 min followed by reperfusion group (I/R-MCAO); permanent middle cerebral artery occlusion group (p-MCAO); permanent middle cerebral artery occlusion in diabetic rat group (Dia p-MCAO). Streptozotocin (40 mg/kg, Sigma, St. Louis, MO, U.S.A.) dissolved in citrate buffer (0.1 mM, pH 4.2) and injected intraperitoneally to induce diabetic symptoms (23). Blood glucose levels were determined using (Accu-Chek-Roche Diagnostics, Mannheim, Germany) and diabetes was defined as fasting blood glucose >300 mg/dL.

### MCAO Surgery

**Figure 1:**
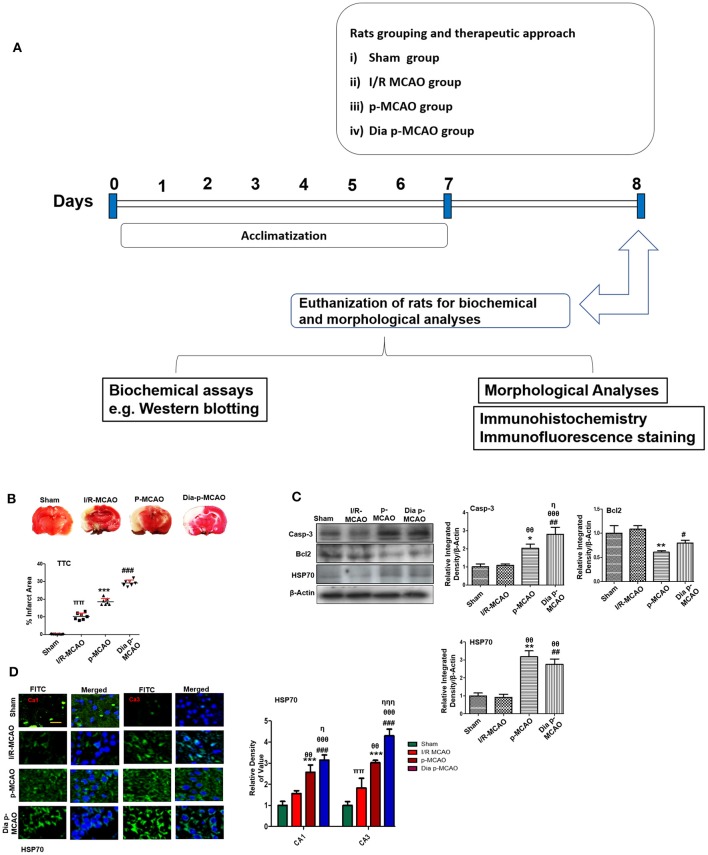
Relative effect of ischemia on brain infarction and cell apoptosis **(A)** Flow chart of experiment. **(B)** Brain coronal sections were stained with TTC, which distinguishes between ischemic and non-ischemic areas, one ways ANOVA followed by *post-hoc* bonferroni multiple comparison test using graph-pad prism-5 software (*n* = 7/group). *p < 0.05, ^#^*p* < 0.05, and ^π^*p* < 0.05 relative to sham group. **(C)** Western blot analysis of Casp-3, Bcl2, HSP70. Densitometric analysis was expressed in relative to β-Actin (*n* = 5/group). *p < 0.05 and ^#^*p* < 0.05 relative to sham group, ^θ^*p* < 0.05 relative to I/R MCAO, ^η^*p* < 0.05 between p-MCAO and Dia p-MCAO. ^θθ^shows significant difference of p-MCAO and Dia p-MCAO to I/R, and its value is *p* < 0.01, ^θθθ^shows significant difference of p-MCAO and Dia p-MCAO to I/R, and its value is *p* < 0.001, ^ππ^shows significant difference of I/R MCAO to sham, and its value is *p* < 0.01, ^ηηη^shows significant difference between p-MCAO and Dia p-MCAO, and its value is *p* < 0.001. **(D)** Representative images of HSP70 immunofluorescence staining in CA1 and CA3 region of hippocampus (*n* = 5/group). Scale bar = 100 μm. *p < 0.05 and ^#^*p* < 0.05 relative to sham group, ^θ^*p* < 0.05 relative to I/R MCAO, ^η^*p* < 0.05 between p-MCAO and Dia p-MCAO. **p < 0.01, ***p < 0.001, ^*##*^*p* < 0.01, and ^*###*^*p* < 0.001.

MCAO procedure were operated as previously described (24–26). Briefly, rats were anesthetized by mixture of xylazine and ketamine (1:3.2, I/P). The body temperatures of rats were maintained at 37 ± 1°C by using blanket and heating lamps. Briefly, a cervical incision was achieved on ventral side, keeping the incision laterally toward right region. The underlying tissues were carefully dissected to locate the right common carotid artery (CCA), and which was further set free from thin vagus nerve run laterally to CCA. The two bifurcating branches of CCA, external and internal carotid artery were identified and set free from surrounding tissues. The thin smaller arteries, occipital artery, and superior thyroid artery arising from external carotid artery were ligated with black silk (6/0) and subsequently pierced. A permanent knot was applied to external carotid artery above the origin of superior thyroid artery near hyoid bone. Moreover, the external carotid artery was cut by a sharp scissor near the bifurcating point, and immediately a thick nylon silk with dimension (3/0) having length 3 cm while keeping the tip of the silk round manually by heat, was inserted from the opening of external carotid artery and advanced further into internal carotid artery to the origin of middle cerebral artery (MCA), whereas a small resistance indicated the occlusion of MCA. Twenty-four hours after occlusion, all animals were killed for sample collection. The sham group was exposed to similar measures but with no nylon insertion. The filament remained in placed in rats undergoing p-MCAO but removed 90 min later after stroke onset in transient ischemic rats (Figure 1A). All ischemic rats were returned to the cages and were observed for 24 h. Ten rats were died during the experiment including 3 from p-MCAO group, 2 from t-MCAO, 5 from Dia + p-MCAO, which we excluded from the study. Various methodologies are being used for stroke induction, broadly classified into craniotomies methods using photothrombosis and electrocoagulation. Both kind of transient and permanent occlusion can be induced by this. A major disadvantage associated with these procedures are large craniotomies and sometimes damage to skin and skull structures. Moreover, the intraluminal method using blue nylon silk is widely used in experimental procedures, though the major limitation associated with this model is subarachnoid hemorrhage due to vessel rupturing and hyperthermia.

### TTC Staining

TTC staining was performed as previously described (27). Briefly, brain tissues were carefully removed and washed with cold PBS. Three to four millimeter-thick coronary sections were cut by using sharp blade from frontal lobe. These coronal slices were incubated in 2% TTC for 10–20 min, until a thorough demarcation was observed for MCAO operated rats; while sham operated rats were stained deep red. ImageJ software was used to quantitatively determine infarcted area by optimizing background of image to threshold intensity and then calculated infarct area from total brain area. To compensate for brain edema, the corrected brain infarction was calculated as follow:Corrected infarct area = left hemisphere area – (right hemisphere area – infarct area).

### Western Blot

For Western blot analysis, the samples were dissolved in lysis buffer (1M Tris–HCI, 5M sodium chloride, 0.5% sodium deoxycholate, 10% sodium dodecyl sulfate, 1% sodium azide, 10% NP-40) (28). The homogenate was sonicated, centrifuged and protein concentration was determined by BioRad protein assay kit (BioRad Laboratories, CA, and USA) according to guidelines provided by manufacturer. Equal amount of protein (i.e., 30 μg per sample) were electrophoresed on 10% SDS-PAGE gels followed by immunoblotting for transferring the protein to poly-vinylidene fluoride (PVDF) membranes (Millipore, Billerica, MA, USA). PVDF was washed in Tris-buffered saline containing 0.1% Tween-20 (TBST) and then incubated with primary antibodies overnight at 4°C. The membranes were then incubated with appropriate secondary antibody, and protein band were detected using an ECL detection reagent according to the manufacturer's instructions (Amersham Pharmecia Biotech, Uppsala, Sweden). The X-ray films were scanned, and optical densities of the bands were analyzed through densitometry using computer-based ImageJ program.

The antibodies used include anti-HSP70 (SC-66048), anti-Bcl2 (SC-2960), anti-Caspase3 (SC-7148), anti-NRF2 (SC-722), anti- HO-1 (SC-136961), anti-p-JNK (SC-6254), anti-JNK (SC-7345), anti-P38 (SC-7972), anti-ERK (SC-135900), anti-NR2a (SC-1468), anti-NR2b (SC-1469), anti-PSD95 (SC-71933), anti-GluR1 (SC-55509), anti-p-GluR1 serine831 (SC-16313), anti SNAP25 (SC-7538), anti-Synaptophysin (SC-17750), anti-BDNF (SC-546), anti-VEGF (SC-7269), anti-p-NF_k_B, anti-TNF (SC-52746), anti-GFAP (SC-33673), anti-COX2 (SC-7951), and anti-β-Actin from (Santa Cruz, Biotechnology, CA, USA) at dilution of 1:1,000. Anti-p-P38 (Cat # 4511), anti-p-ERK (Cat # 8544), and anti-p-GluR1serine845 (Cat # 8084) were purchased from Cell Signaling.

### Immunohistochemistry

Brain tissues were fixed in 4% paraformaldehyde and embedded in paraffin, and 4 μm coronary sections were cut using a rotary microtome (29). Tissue sections on coated slides were de-paraffinized, with three different absolute xylenes and were rehydrated with ethyl alcohol [from 100% (absolute) to 70%]. The slides were rinsed with distilled water and immersed in 0.01 M phosphate-buffered saline (PBS) for 10 min. The slides were processed for antigen retrieval step using a heat method. The slides were allowed to cool and washed with PBS twice times. After antigen retrieval, the slides were incubated with 3% hydrogen peroxidase to quench endogenous peroxidase and were subsequently blocked with 5% serum depending upon the sources of secondary antibodies used. After blocking, the slides were incubated overnight in mouse anti-VEGF (SC-7269, Santa Cruz Biotechnology) at 1:100 dilution. followed by treatment with appropriate biotinylated secondary antibodies for 2 h and successively with ABC reagents (Santa Cruz Biotechnology) for 1 h at room temperature. The sections were washed with PBS and stained in 3, 3′-Diaminobenzidine tetrahydrochloride solution; they were then washed with distilled water, dehydrated in graded ethanol (70, 95, and 100%), fixed in xylene, and cover-slipped by a mounting medium. Immunohistochemical results were analyzed by a light microscope (Olympus, Japan), which was connected to a digital photomicroscopy system. ImageJ software was used to quantitatively determine hyperactivated VEGF, by optimizing background of image to threshold intensity and analyzing cytoplasmic VEGF positive cells at the same threshold intensity for all groups and was expressed as the relative integrated density of the samples relative to the sham.

### Fluoro-Jade B Staining

The slides were immersed in a solution of 1% sodium hydroxide, graded ethanol, and then in distilled water. Slides were transferred into a coplin jar and were washed with 0.06% potassium permanganate solution for 10 min. The slides were rinsed with distilled water, and then transferred to a 0.01% Fluoro-Jade B solution containing 0.1% acetic acid. The slides were then washed with distilled water and air dried. The slides were incubated with DAPI and cover slipped with non-fluorescent mounting media and photographed. ImageJ software was used to quantitatively determine fluorescence intensity of hippocampus/total area for all groups. The immunofluorescence intensity was expressed as the relative integrated density of the samples relative to the sham.

### Immunofluorescence Analysis

After de-paraffinization of sections, the slides were autoclaved in 0.1M sodium citrate pH 6 for antigen retrieval step (30). The slides were allowed to cool and washed with PBS twice times. Slides were incubated with 5% normal serum depending upon the source of secondary antibody used. The slides were incubated with primary antibodies at 4°C overnight (HSP70, 8-oxoguanine p-JNK, COX2, GFAP) from Santa Cruz Biotechnology at 1:100 dilution. Next morning, after washing with PBS, fluorescent labeled secondary antibodies (Santa Cruz Biotechnology) as 1:50 dilution were used for signal amplification in dark chamber, followed by mounted with UltraCruz mounting medium (Santa Cruz Biotechnology). The slides were pictured with confocal scanning microscopes (Flouview FV 1000, Olympus, Japan) and fluorescence intensity was quantitatively analyzed by ImageJ and expressed as the relative integrated density of the samples relative to the sham.

### Statistical Analysis

Western blot bands and morphological data were quantified using ImageJ software (Image J 1.30; // https://imagej.nih.gov/ij/) and analyzed by GraphPad Prism 5 software. Data were presented as means ± SD. Data were analyzed by one ways ANOVA followed by *post-hoc* Bonferroni Multiple Comparison test using graph-pad prism-5 software. Symbols * or # or π or θ or η represent significant difference values *p* < 0.05. Symbols *, # and π shows significant difference relative to sham. Symbol θ shows significant difference relative to I/R MCAO, η shows significant difference between p-MCAO and Dia p-MCAO.

## Results

### Evaluation of Neurodegeneration in Ischemic Models

Cerebral ischemia brings robust neuronal changes in the core brain areas where dramatic blood flow reduction caused irreversible cell death. Neuronal apoptosis could be seen after MCAO in the ischemic penumbra or peri-infarct zone, where blood flow is less severely reduced. Sham group showed no infarction, while extensive infarction was observed for different MCAO operated rats (Figure 1B). A significant inter group variability was observed, whereas Dia p-MCAO and p-MCAO group showed extensive infarction, than I/R MCAO group relative to sham group. Further calculations of corrected % infarcted area were higher for Dia p-MCAO (28.35 ± 4.3) than p-MCAO (22.71 ± 3.9 %) and I/R-MCAO (11.4 ± 3.5). Unlikely no noticeable infarction was observed at hippocampus in any ischemic group (Figure 1B). The apoptotic (Caspase-3) and anti-apoptotic protein marker (Bcl2) expression were also evaluated to validate the relative expression in different ischemic models (Figure 1C). No significant alterations were noticed in I/R group for these markers, contrary to p-MCAO (Figure 1C). Furthermore, Heat shock proteins (HSP) are physiological sensors that delineate extent of injury in core, penumbral and peri-infarct tissue. We therefore examined HSP70 expression by western blot. The results showed elevated expression of HSP70 in permanent and diabetic ischemic group relative to sham (Figure 1C), implies that peri-infarct waves spread more sporadically in p-MCAO than I/R MCAO. Moreover, western blot results were also validated by immunofluorescence findings (Figure 1D) in CA1 and CA3 region of hippocampus.

### NMDA Receptor Signaling

**Figure 2:**
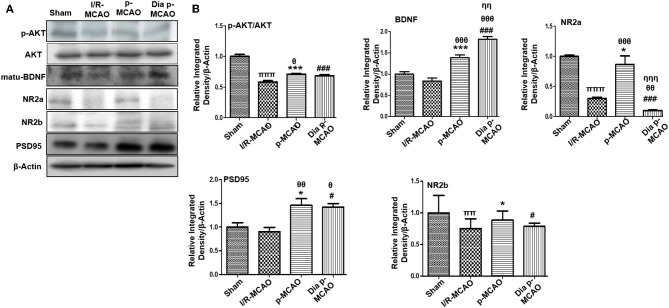
Effect of ischemia on NMDA receptor related molecule changes **(A)** Representative Western blot of p-AKT, AKT, matu-BDNF, NR2a, NR2b, PSD95. **(B)** Histograms indicating comparative expression of various glutamate receptors in hippocampus. Densitometric analysis was expressed in relative to β-Actin (*n* = 5/group). *p < 0.05, ^#^*p* < 0.05, and ^π^*p* < 0.05 relative to sham group. ^θ^*p* < 0.05 relative to I/R MCAO, ^η^*p* < 0.05 between p-MCAO and Dia p-MCAO. ***p < 0.001 and ^*###*^*p* < 0.001. ^ππ^shows significant difference of I/R MCAO to sham, and its value is *p* < 0.01, ^πππ^shows significant difference of I/R MCAO to sham, and its value is *p* < 0.001, ^θθ^shows significant difference of p-MCAO and Dia p-MCAO to I/R, and its value is *p* < 0.01, ^θθθ^shows significant difference of p-MCAO and Dia p-MCAO to I/R, and its value is *p* < 0.001, ^ηη^shows significant difference between p-MCAO and Dia p-MCAO, and its value is *p* < 0.01, ^ηηη^shows significant difference between p-MCAO and Dia p-MCAO, and its value is *p* < 0.001.

Several reports reiterated NMDA excitotoxicity to be the fundamental cause of ischemic induced neuronal damage. Synaptic NR2a activation mediates NR2a/AKT signaling kinases, linked to CREB activation for neuronal survival (31). BDNF also demonstrated to activate several kinases including AKT (32). Our result suggested attenuated expression of NR2a and NR2b receptor subunit expression. These results further provide support to the previous observations, delineating MCAO induced calpain degradation with concurrent lower expression of neuronal survival pathways downward of NR2a (33). Moreover, calpain induced degradation and decreased expression of full length NR2a in MCAO can be reversed by increased interaction of PSD95/NR2a (34). NR2a and NR2b were differentially expressed in these ischemic models, whereas NR2a significantly downregulated in I/R and dia p-MCAO (Figure 2), compare to p-MCAO. Unlikely, for NR2b, I/R group showed maximum cleavage than other ischemic group. The results of PSD95 is in line with NR2a expression for I/R group and p-MCAO, as PSD95 did not change in I/R, which may suggest increase susceptibility of NR2a to cleavage, while hyperexpression of PSD95 in p-MCAO may hypothetically be explained for promoting NR2a downward survival pathway.

### Expression of MAPK Family Protein in Ischemic Models

**Figure 3:**
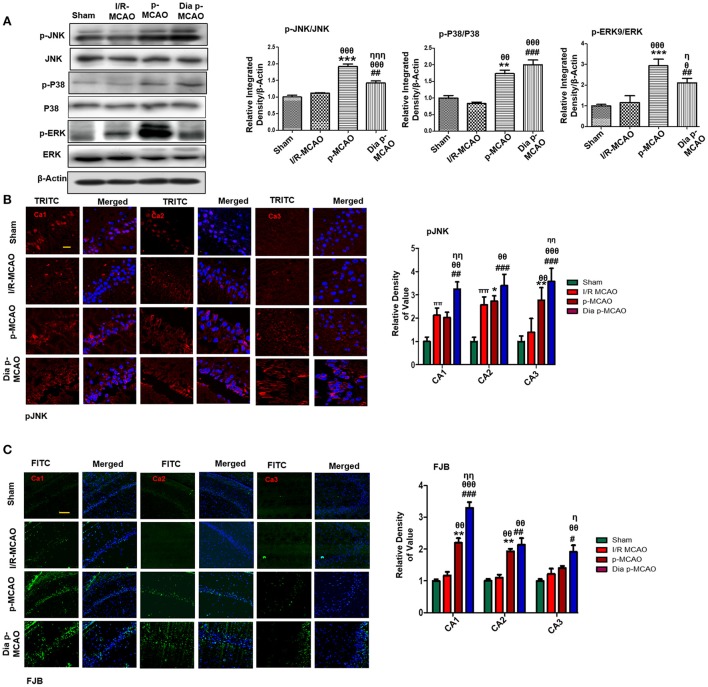
Ischemia induced cell stress and death related signaling changes **(A)** Representative Western blots of p-JNK, JNK, p-P38, P38, p-ERK, ERK. Densitometric analysis was expressed in relative to β-Actin (*n* = 5/group). **(B)** Representative images of p-JNK immunoreactivity in CA1, CA2, and CA3 regions of hippocampus in I/R, p-MCAO and Dia p-MCAO group (*n* = 5/group). scale bar = 100 μm. **(C)** Representative images of FJB histochemistry showing apoptotic cells; scale bar = 30 μm. Significant neuronal apoptosis in CA1, CA2 and CA3 region of hippocampus was observed in p-MCAO and Dia p-MCAO group (*n* = 5/group). ^θ^*p* < 0.05 relative to I/R MCAO, ^η^*p* < 0.05 between p-MCAO and Dia p-MCAO. *p < 0.05, ^#^*p* < 0.05, and ^π^*p* < 0.05 relative to sham group. ***p < 0.001, **p < 0.01, ^*###*^*p* < 0.001, and ^*##*^*p* < 0.01. ^θθ^shows significant difference of p-MCAO and Dia p-MCAO to I/R, and its value is *p* < 0.01, ^θθθ^shows significant difference of p-MCAO and Dia p-MCAO to I/R, and its value is *p* < 0.001, ^ηη^shows significant difference between p-MCAO and Dia p-MCAO, and its value is *p* < 0.01, ^ηηη^shows significant difference between p-MCAO and Dia p-MCAO, and its value is *p* < 0.001.

Several studies demonstrated association of P38 MAPK, and c-Jun-N-terminal kinases (JNKs) to apoptotic cell death. Western blot analysis demonstrated elevated expression of activated p38 and JNK (p-JNK) in permanent and in diabetic ischemic group compared to sham and I/R operated group (Figure 3A). Furthermore, immunohistochemistry results were validated by western blot findings for p-JNK in permanent and diabetic ischemic models (Figure 3B). Both neuroprotective and neurotoxic effects are attributed to ERK activation (35, 36). ERK is linked to neuronal injury in ischemic brain model (36). We found hyperactivated ERK in permanent and in diabetic ischemic group compared to sham operated group (Figure 3A).

Moreover, Fluoro-Jade B (FJB) staining was performed to examine apoptotic cell death. Significant numbers of FJB-positive cells were noticed in CA1, CA2, and CA3 region of hippocampus in permanent and diabetic ischemic group (Figure 3C). The sham and I/R group did not exhibit significant positive staining. The FJB data is parallel to Figure 1A, where no significant changes were observed in I/R group for Caspase-3 and Bcl2.

### Glutamate Neurotransmission and Axonal Degeneration

**Figure 4:**
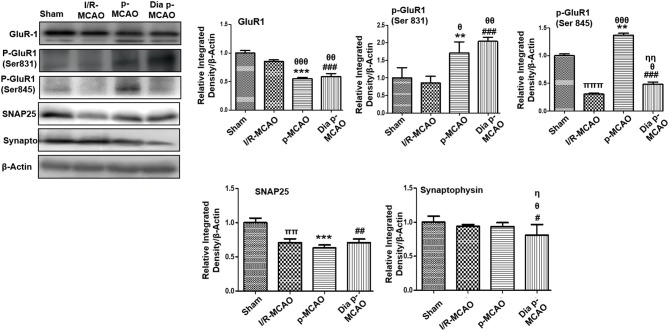
Expression of glutamate neurotransmission and synaptic changes in various ischemic models western blot analysis of GluR1, p-GluR1 (Ser845, Ser831), SNAP25, Synaptophysin. Densitometric analysis was expressed in relative to β-Actin (*n* = 5/group). ^θ^*p* < 0.05 relative to I/R MCAO, ^η^*p* < 0.05 between p-MCAO and Dia p-MCAO. *p < 0.05, ^#^*p* < 0.05, and ^π^*p* < 0.05 relative to sham group. ***p < 0.001, **p < 0.01, ^*###*^*p* < 0.001, and ^*##*^*p* < 0.01. ^θθ^shows significant difference of p-MCAO and Dia p-MCAO to I/R, and its value is *p* < 0.01, ^θθθ^shows significant difference of p-MCAO and Dia p-MCAO to I/R, and its value is *p* < 0.001, ^ππ^shows significant difference of I/R MCAO to sham, and its value is *p* < 0.01, ^πππ^shows significant difference of I/R MCAO to sham, and its value is *p* < 0.001, ^ηη^shows significant difference between p-MCAO and Dia p-MCAO, and its value is *p* < 0.01.

AMPA receptor activation is triggered by glutamate accumulation following cerebral ischemia. In line with previous reports, we observed significantly down expression of glutamate receptor (AMPA, GluR-1) receptor in p-MCAO groups compare to I/R operated group in hippocampus (Figure 4). We also studied the phosphorylation of AMPA (GluR1) at serine 831 and 845. A marked increase at serine 831 were observed in p-MCAO groups (Figure 4), while no change was observed in I/R group. Moreover, serine 845 was found with aberrant expression in permanent MCAO group, and with decrease expression in diabetic and I/R (Figure 4).

Synaptosomal associated protein 25 (SNAP-25), and synaptophysin acts as synaptic marker for neuronal differentiation. To examine the detrimental effect of ischemic damage on synaptic proteins following cerebral ischemia, we studied the expression of synaptophysin, and synaptosomal-associated protein-25 (SNAP-25) (Figure 4). It was observed that expression of SNAP-25 decreased in all ischemic operated animals (Figure 4), while no significant changes were observed in expression of synaptophysin except for dia p-MCAO (Figure 4).

### Effect on Inflammatory Markers

**Figure 5:**
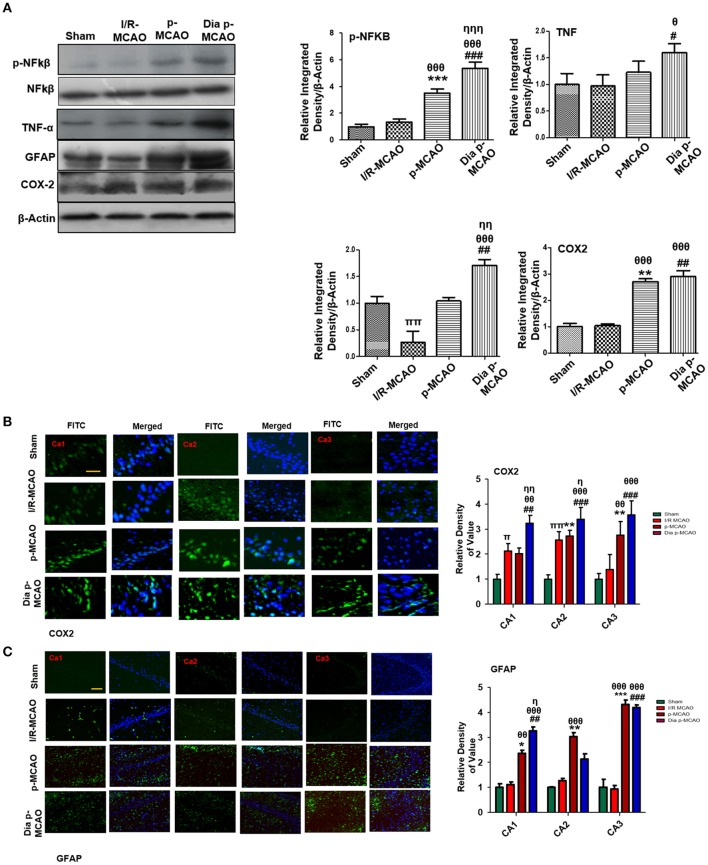
Effect of ischemia on inflammatory related molecules in the hippocampus **(A)** Western blot analysis of p-NF_κ_B, NF_κ_B, TNF_α_, GFAP, and COX2. Densitometric analysis was expressed in relative to β-Actin (*n* = 5/group). **(B)** Representative photos of immunohistochemistry for COX2, scale bar = 100 μm, and **(C)** GFAP; scale bar = 30 μm, (*n* = 5/group). The CA1, CA2, and CA3 segments in p-MCAO and Dia p-MCAO showing elevated expression of COX2 and GFAP. ^θ^*p* < 0.05 relative to I/R MCAO, ^η^*p* < 0.05 between p-MCAO and Dia p-MCAO. *p < 0.05, ^#^*p* < 0.05, and ^π^*p* < 0.05 relative to sham group. ***p < 0.001, **p < 0.01, ^*###*^*p* < 0.001, and ^*##*^*p* < 0.01. ^θθ^shows significant difference of p-MCAO and Dia p-MCAO to I/R, and its value is *p* < 0.01, ^θθθ^shows significant difference of p-MCAO and Dia p-MCAO to I/R, and its value is *p* < 0.001, ^ηη^shows significant difference between p-MCAO and Dia p-MCAO, and its value is *p* < 0.01, ^ηηη^shows significant difference between p-MCAO and Dia p-MCAO, and its value is *p* < 0.001, ^ππ^shows significant difference of I/R MCAO to sham, and its value is *p* < 0.01.

Reports have consistently supported TLR4 activated downstream inflammatory mediators such as p-NF-κB, iNOS, and COX2 in ischemic brain injury. To evaluate whether these mediators could also be activated in hippocampus in different ischemic models, we performed western blotting. Moreover, the activation of TLR4 is linked to many downstream effects including activation of p-NF-κB, iNOS, and COX2. Western blot results showed elevated expression of these mediators in permanent ischemic brain (Figure 5). Furthermore, the western blot data was validated by immunostaining findings for COX2 (Figure 5B). Activation of p-NF-κB encodes the bulk of inflammatory mediators that exacerbate ischemic brain injury. Ischemic stroke is characterized by reactive gliosis in which the resident microglia and astrocytes assume a characteristics cellular appearance to mediate progression of ischemic injury. These activated hypertrophic cells work as resident machinery for generating inflammatory mediators. We investigated the expression of astrocytes in ischemic hippocampus (GFAP reactive cells) following 24 h of permanent ischemia (Figure 5C). Immunofluorescence results revealed a significant increase in the number of GFAP reactive cells in (MCAO) compared to sham (Figure 5C).

### Effect on Oxidative Stress

**Figure 6:**
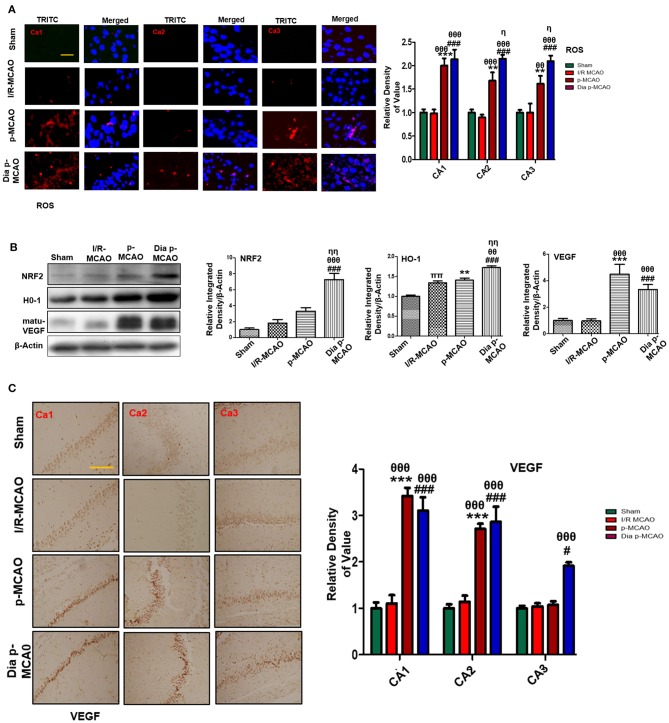
Effect of ischemia on oxidative distress **(A)** Representative immunofluorescence images of 8-oxoguanine staining indicate comparative expression of ROS in various CA1, CA2, and CA3 region of hippocampus (*n* = 5/group, Scale bar = 100 μm). **(B)** Western blot analysis of NRF2, HO-1, matu-VEGF. Densitometric analysis was expressed in relative to β-Actin (*n* = 5/group) **(C)** Representative images of VEGF immunohistochemistry; scale bar = 50 μm. The CA1, CA2, and CA3 segments in p-MCAO and Dia p-MCAO showing elevated expression of VEGF (*n* = 5/group). ^θ^*p* < 0.05 relative to I/R MCAO, ^η^*p* < 0.05 between p-MCAO and Dia p-MCAO. *p < 0.05, ^#^*p* < 0.05, and ^π^*p* < 0.05 relative to sham group. ***p < 0.001, **p < 0.01, and ^*###*^*p* < 0.001. ^θθ^shows significant difference of p-MCAO and Dia p-MCAO to I/R, and its value is *p* < 0.01, ^θθθ^shows significant difference of p-MCAO and Dia p-MCAO to I/R, and its value is *p* < 0.001, ^ηη^shows significant difference between p-MCAO and Dia p-MCAO, and its value is *p* < 0.01, ^ππ^shows significant difference of I/R MCAO to sham, and its value is *p* < 0.01.

To further estimate oxidative stress in hippocampus, we used fluorescent dye 8-oxoguanine as oxidative stress marker. Our result demonstrated elevated expression of 8-oxoguanine particularly in diabetic ischemic group (Figure 6A). Moreover, the ubiquitously expressed nuclear erythroid 2-related factor-2 (Nrf2) is an endogenous antioxidant enzyme, translocate to nucleus for stimulating several downstream antioxidant proteins such as HO-1, superoxide dismutase (SOD) and glutathione (GSH) (37). Western blot results demonstrated elevated expression of Nrf2 and HO-1 in diabetic group (Figure 6B). VEGF is specific endothelial growth factor, implicated both in disease and normal condition. Several studies demonstrated linkage of reduced VEGF in neurodegeneration (38). Moreover, VEGF signaling, and angiogenesis are modulated by ROS formation (39). Previous studies suggested the pro-angiogenetic effect of NRF2 and down regulation of NRF2 abrogated NRF2 induced vascular sprouting (40).

To observe the expression of these growth factors in hippocampus at different ischemic intervals, we performed western blot analysis 24 h after MCAO (Figure 6B). Moreover, immunohistochemistry results further validate western blot findings for VEGF in permanent ischemic models (Figure 6C).

## Discussion

Stroke is the most devastating human health condition. Different kind of animal models are used in the laboratory to mimic human ischemic stroke with addition of different occlusion periods. Necrotic cell death primarily occurs in ischemic core, while apoptotic cell death mostly targets the penumbral tissues. The demarcation between these tissues largely depends on time of occlusion. Several studies confirmed apoptotic cell death in hippocampus after 24 h of ischemia in different rat strain (15, 41, 42). Our results are parallel to previous published reports, found apoptotic cell death in hippocampus after 24 h of ischemia (41, 43). Moreover, these authors found no changes in Caspase and Bcl-2 expression in hippocampus using I/R model. Likely, we found no changes in I/R group, but these proteins were significantly disturbed in diabetic and permanent ischemic group.

In the current manuscript, we found significant differences in protein expression among t-MCAO, p-MCAO, and diabetic p-MCAO at hippocampus. Our result suggested that peri-infarct localization can extend to hippocampus after 24 h of p-MCAO, as shown by HSP70 expression (Figures 1B,C). Hyperglycemics condition exacerbate MCAO induced brain damage by compromising vascular permeability and exacerbating neuronal toxicity. Likely, the protein expression and morphological findings in this study showed severe disturbances in diabetic and permanent ischemic group than t-MCAO. Overexpressed HSP70 indicates compromised protein synthesis due to reduced blood supply in hippocampus (44), in addition it reflects potential tissue damage (45). It is previously demonstrated that protein synthesis in penumbral and peri-infarct tissue is inhibited within hours after ischemia (46, 47). Induced HSP70 could also serve as endogenous protector to cope against denatured protein induced by ischemia (48).

Several reports consistently pointed out glutamate (Glu) excitotoxicity to be the fundamental hallmark of ischemic induced neurodegeneration (49). Previous results demonstrated that glutamate neurotoxicity can be observed within 2–4 h of ischemic occlusion (50, 51). In I/R MCAO models, glutamate restores to basal level quickly. However, this alteration in glutamate level can be observed for a prolonged period due to collapse of exchange pumps in p-MCAO models (51, 52). The role of NMDA in ischemic brain injury is not clearly known due to conflicted results (49, 53, 54). It was suggested that NR2a subunit of NMDA lead to neuronal survival while NR2b is linked to neuronal apoptosis (33, 49). Furthermore, calpain is activated by ischemic injury, which degrades a large array of molecules including NMDA receptor subunits NR2a and NR2b (55). Interestingly, our results found different response patterns of NR2a and NR2b and the downstream signaling of AKT, suggesting that NMDA receptors may underlie distinct processes of glutamate transmission.

Ischemic stroke rigorously disrupts synaptic networks in hippocampus tissue. A transient ischemic occlusion is responsible for closure of about 30% synapses in hippocampus region (56). Some reports also suggested synaptic remolding after ischemic damage in hippocampus with correspondingly increased synapse formation (57, 58). AMPA receptor activity is controlled by several mechanisms including phosphorylation and calpain mediated cleavage of GluR1 subunit (59). In line with previous reports, we observed down expression of GluR1 in ischemic hippocampus. Phosphorylation at serine 845 is involved in the translocation of AMPA to neuronal membrane and such AMPAR phosphorylation can boost synaptic plasticity for learning and memory (60). Ischemic brain injury impairs trafficking of AMPA by down regulating (GluR1 serine 845) phosphorylation. Synaptophysin and SNAP-25 level was compromised in ischemic brain injury significantly in permanent and diabetic ischemic group similar to previous observation (61). Moreover, PSD95 showed high expression in permanent and diabetic ischemic as observed previously (56, 62).

Neurotropic factors are linked to neuroplasticity which accelerates neuronal repairment during ischemic brain injury. Brain derived neurotrophic factor (BDNF), vascular endothelial growth factor (VEGF) are categorized as neurotrophins having important pleiotropic effects on brain structuring. Studies suggested that BDNF and VEGF governs neurogenesis and improve functional outcomes after ischemic stroke (63, 64). In line with previous research, our results demonstrated hyperactive VEGF and BDNF in hippocampus. The induction of VEGF after cerebral ischemia represents inherent defense mechanism, whereby expressed VEGF leads to vascularization and sprouting of blood vessel to cope with the severe demand of energy.

Release of inflammatory proteins further exacerbate ischemic stroke injury by several mechanisms, such as activation of Toll like receptor (TLR-4) on glial cells stimulates stress kinases like (JNK and P38-MAPK) (65). Activation of these pathways triggers mitochondrial apoptotic pathway while inhibiting these kinases attenuates inflammatory cytokines (66). Cytokines are integral component of inflammation and produced largely by activated microglia, astrocytes, and neurons immediately within hour after ischemic injury. Twenty-four hours after permanent ischemia, higher expression of NF-_K_B can trigger iNOS, COX-2 production; both are toxic mediators of inflammatory cascade (67, 68). Besides its role in neuron inflammation, NF-_K_B is also involved in oxidative stress via the activation of NRF2, a ubiquitous transcription factor that modulates the expression of various oxidant and antioxidant process related proteins, such as HO-1 and superoxide dismutase (SOD). Our results demonstrated elevated expression of Nrf2 and HO-1 in all three models, showing that the exacerbating effects of prolonged ischemic time and diabetic conditions.

In conclusion, our result suggests that hippocampus is adversely affected by ischemic injury after 24 h of intraluminal occlusion using 3/0 nylon silk. We noticed ischemic driven alteration in protein expression and histological changes in various segments of hippocampus. Furthermore, the most two commonly used MCAO models exhibited tremendous differences in terms of neuronal cell loss and neurological deficits, glutamate excitotoxic related signaling, synaptic transmission markers, neuron inflammatory and oxidative stress molecules. These differences may reflect the variations in different models, which may provide valuable information for mechanistic and therapeutic inconsistences as experienced in both preclinical models and clinical trials. More interestingly, the pathological profiles of p-MCAO in diabetic rats showed that a wide array of molecular processes are involved in the devastating effects of hyperglycaemia other than proposed changes of oxidative stress and neuron inflammation.

## Data Availability Statement

All data generated or analyzed during this study are included in this published article.

## Ethics Statement

Adult male Sprague-Dawley rats weighing 200–230 g (8–9 weeks) were purchased from Guangdong Medical Laboratory Animal Center, China. The experimental animals were housed at Laboratory Animal Research Center, Peking University Shenzhen Graduate School, under 12 h light/12 h dark cycle at 18–22°C and had free access to diet and tap water throughout the study. The experimental procedures were set in such a way to minimize rats suffering. All experimental procedures were carried out according to the protocols approved by Institutional Animal Care and Use Committee of Peking University Shenzhen Graduate School. We did not use any blind allocation or randomization of rats, instead we adhered the criteria to keep similar weight animal to same group under the same experimental condition.

## Author Contributions

FS, AZ, and LK review literature and feasibility. FS, TL, GL, and XY performed surgery, western blot, and morphological experiments. FS, AZ, LK, SK, FL, and HY performed data analysis. HY, A-UK, and PK supported the study. FS and SL designed study and wrote the manuscript. YJ and SL reviewed and approved the manuscript, and held all the responsibilities related to this manuscript. All authors reviewed and approved the manuscript.

### Conflict of Interest

The authors declare that the research was conducted in the absence of any commercial or financial relationships that could be construed as a potential conflict of interest.

## Abbreviations

MCAO: middle cerebral artery occlusion
I/R: ischemic reperfusion
Casp-3: caspase 3
HSP70: heat shock protein
BDNF: brain derived neurotrophic factor
NMDAR: N-methyl-d-aspartate receptor
TTC, 2, 3: 5-triphenyltetrazolium chloride
BCA: bicinchoninic acid
PVDF: poly-vinylidene fluoride
TBST: tris-buffered saline containing 0.1% Tween-20
CST: cell signaling technology
DAB, 3: 3′-diaminobenzidine tetra hydrochloride
SNAP-25: synaptosomal associated protein 25
GSK-3β: glycogen synthase kinase-3β
Glu: glutamate
BDP: break down products
PSD95: post-synaptic density protein
NR2a: N-methyl D-aspartate 2A
NR2b: N-methyl D-aspartate 2b
ERK: extracellular-signal-regulated kinase
FJB: Fluoro-Jade B
Synapto: synaptophysin
NF_K_B: nuclear factor kappa-light-chain-enhancer of activated B cells
TNFα: tumor necrosis factor alpha
COX2: cyclooxygenase
ROS: reactive oxygen species
GFAP: glia fibrillary acid binding protein
VEGF: vascular endothelial growth factor.
